# Supplementary material for: Cytological, Biochemical, and Transcriptomic Analyses of a Novel Yellow Leaf Variation in a Paphiopedilum (Orchidaceae) SCBG COP15
Source: Genes (Basel). 2021 Dec 28;13(1):71. doi: 10.3390/genes13010071 (PMC8775194; doi:10.3390/genes13010071)
Supplement: Supplementary file 1 [file genes-13-00071-s001.zip › Supplementary Figures S1-S6-Revised.pdf]

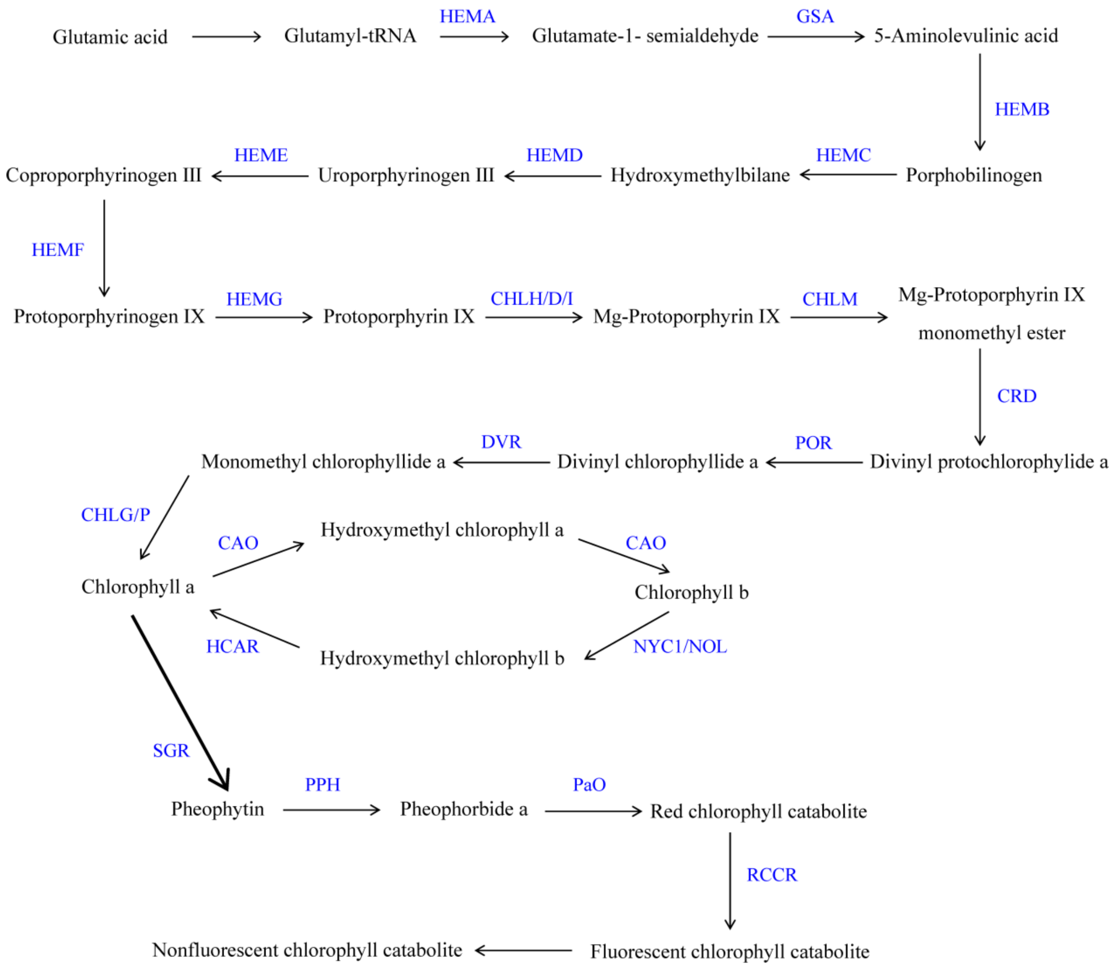

Figure S1. Schematic representation of Chl metabolic pathways in higher plants.

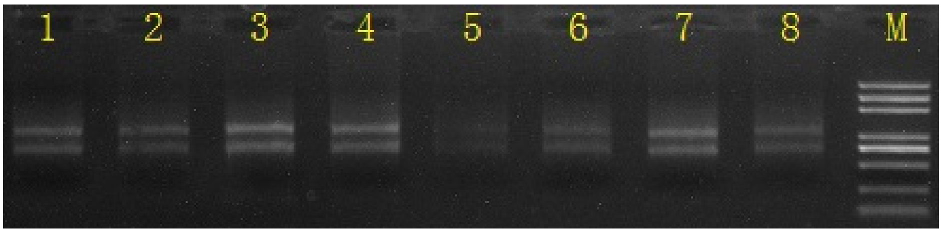

Figure S2. The quality of RNA used for qPCR assessed by agarose gel electrophoresis. 1–4: yellow leaves, 5–8: green leaves.

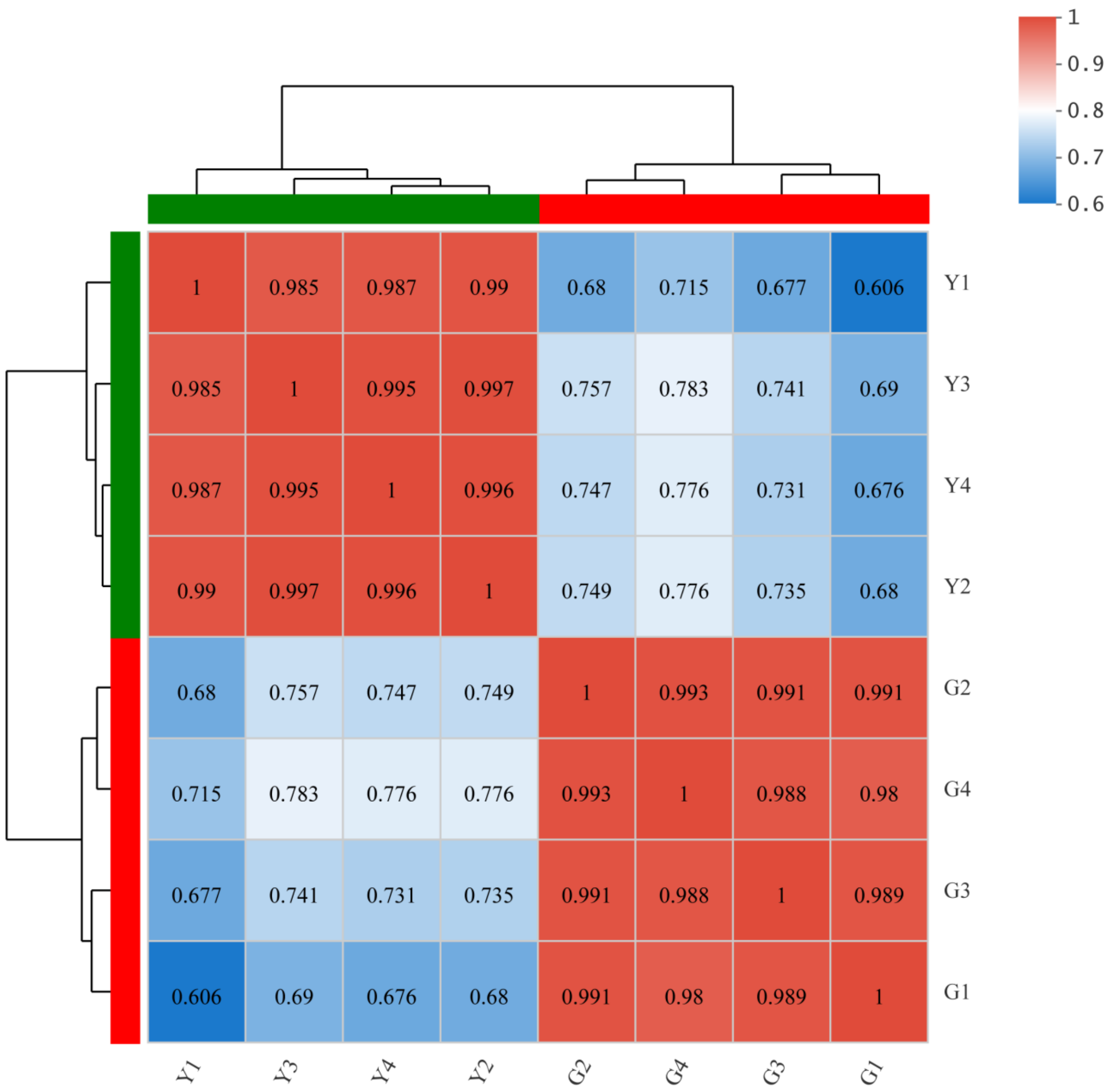

Figure S3. Correlation indices between different samples. Y1-Y4, yellow leaves; G1-G4, green leaves.

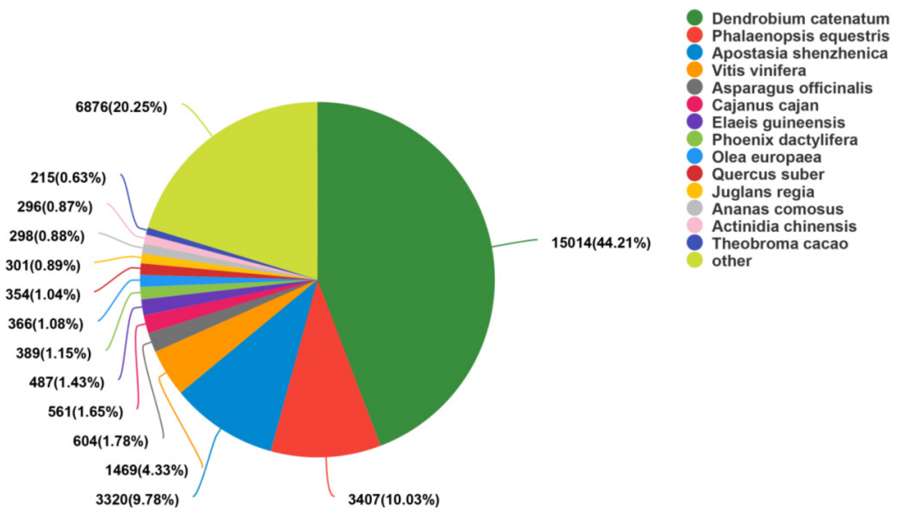

Figure S4. Species distribution of NR annotation. The most abundant annotated species are *D. catenatum* (15,014 unigenes, 44.21%), *Phalaenopsis equestris* (3407 unigenes, 10.03%,) and *Apostasia shenzhenica* (3320 unigenes, 9.78%), which all belong to the orchid family.

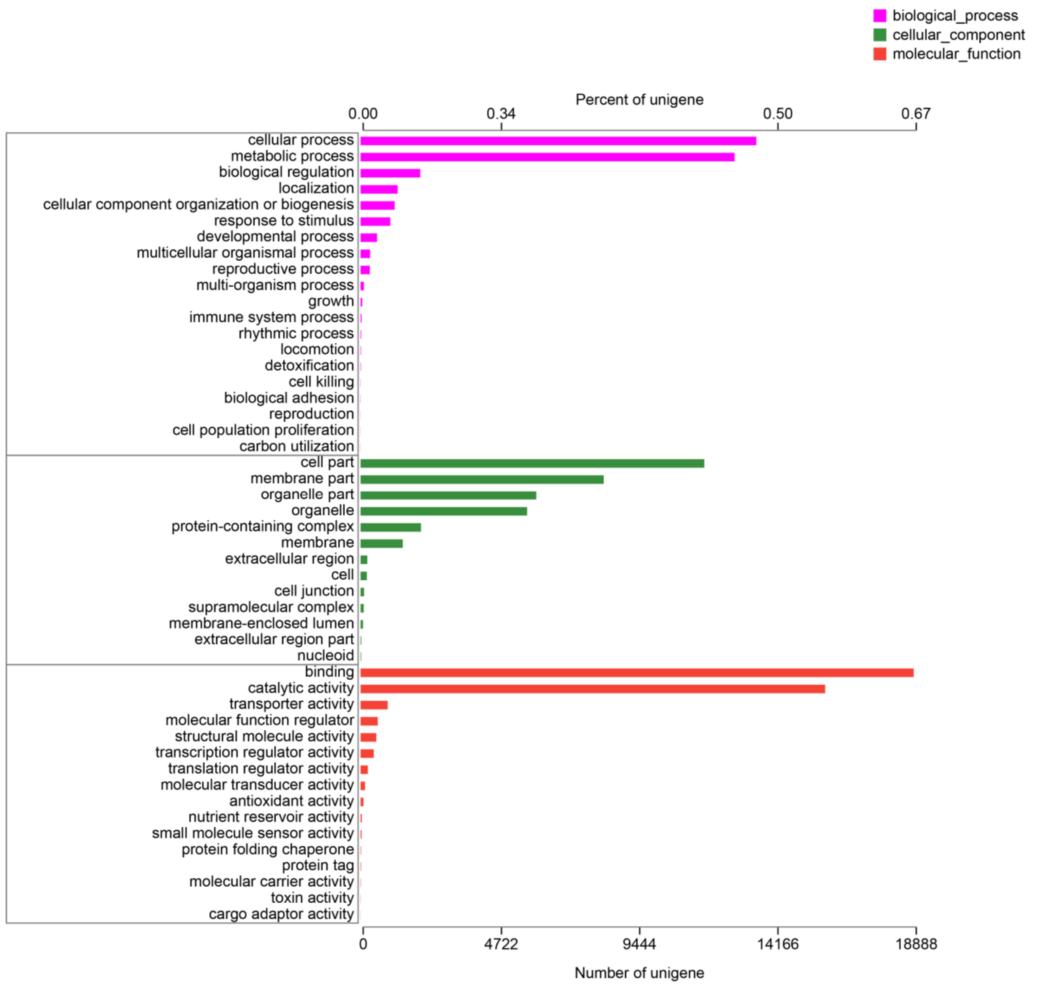

Figure S5. GO functional annotation of all unigenes. The most abundant functions in terms of biological processes are cellular processes and metabolic processes. In terms of cellular components, the most abundant unigenes are related to cell parts and membrane parts, and in terms of molecular function the most abundant unigenes are related to binding and catalytic activity.

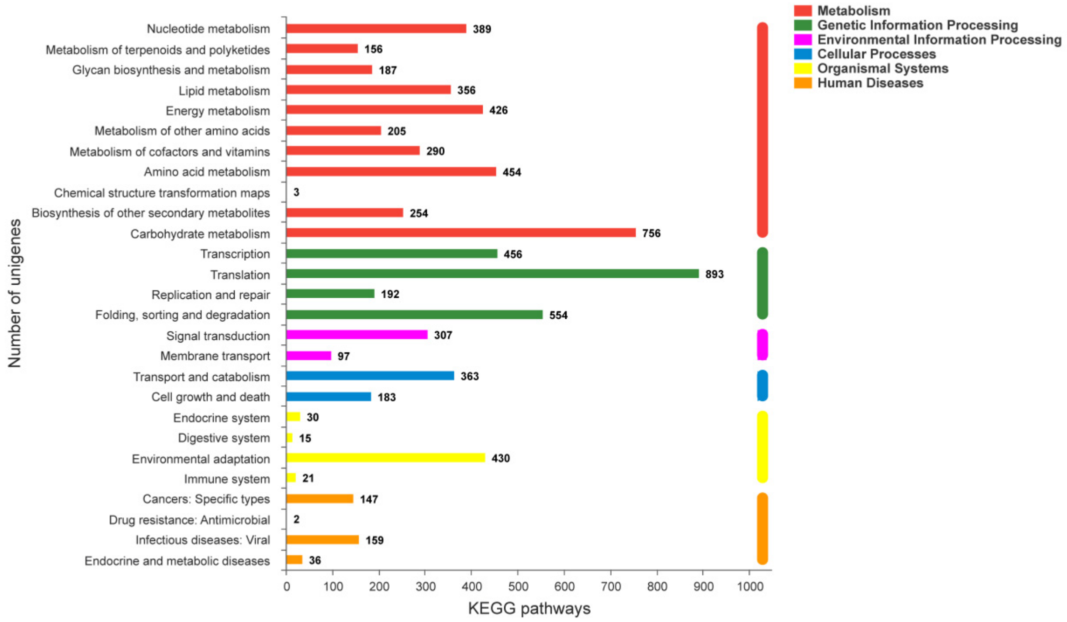

Figure S6. KEGG annotation of all unigenes.
